# Burnout of Physicians, Pharmacists and Nurses in the Course of the COVID-19 Pandemic: A Serbian Cross-Sectional Questionnaire Study

**DOI:** 10.3390/ijerph18168728

**Published:** 2021-08-18

**Authors:** Biljana Jakovljevic, Katarina Stojanovic, Tamara Nikolic Turnic, Vladimir Lj. Jakovljevic

**Affiliations:** 1 Academy for Applied Studies, The College of Health Studies, 11070 Belgrade, Serbia; bilja_boca@yahoo.com; 2 Department of Pharmacy, Faculty of Medical Sciences, University of Kragujevac, Svetozara Markovića 69, 34000 Kragujevac, Serbia; mimiks97@yahoo.de (K.S.); tnikolict@gmail.com (T.N.T.); 3 Department of Physiology, Faculty of Medical Sciences, University of Kragujevac, Svetozara Markovića 69, 34000 Kragujevac, Serbia; 4 Department of Human Pathology, 1st Moscow State Medical, University IM Sechenov, 8 Trubetskaya Street 2, 119991 Moscow, Russia

**Keywords:** COVID-19, health personnel, burnout, Serbia

## Abstract

This research was a prospective, cross-sectional observational study of 128 health workers in the central part of the Republic of Serbia. The study surveyed health workers (physicians, pharmacists and nurses) who worked during peaks of the COVID-19 pandemic in the Republic of Serbia in June and November 2020. The Maslach Burnout Survey for Medical Personnel addresses three scales: (a) emotional exhaustion (EE) measures feelings of being emotionally overextended and exhausted by one’s work; (b) depersonalization (DP) measures an unfeeling and impersonal response toward recipients of one’s service, care treatment, or instruction; and (c) personal accomplishment (PA) measures feelings of competence and successful achievement in one’s work. Linear regression and the chi-square test were used to test a relationship between the input variables (x) and the single output variable (y). We can conclude that most health workers had a high degree of emotional exhaustion, but also a low degree of depersonalization and a high degree of sense of personal achievement. Nurses and physicians had similar answers on the pandemic during their work, but pharmacists had different answers.

## 1. Introduction

Burnout syndrome is defined as the result of chronic stress in the workplace that has not been successfully resolved. It is characterized by three dimensions: a feeling of exhaustion or loss of energy; increased mental distance from the work done or feelings of negativity or cynicism about one’s work; and a sense of inefficiency and lack of achievement [1]. Globally, 30–50% of clinicians have burnout symptoms [2]. The syndrome is diagnosed using various questionnaires, among which the Maslach Burnout Inventory (MBI) is considered the gold standard [3]. If we look only at health care workers and literature data, the prevalence of burnout syndrome is highest among young employees, including physicians who perform risky procedures and nurse-technicians whose duties vary significantly, even on a daily basis, depending on the health care units in which they can be deployed [4,5]. Burnout among physicians has garnered significant attention because of the negative impact it renders on patient care and medical personnel. Physicians who had high burnout levels reportedly committed more medical errors. Stress management programs that range from relaxation to cognitive-behavioral and patient-centered therapy have been found to be of utmost significance when it comes to preventing and treating burnout [6]. Heavy workloads and long hours make stress management a critical skill for pharmacists. A recent study of practicing pharmacists found that more that 68% experienced job stress and role overload [7], and in nursing burnout and somatization is expected. A previous study observed 1363 nurses employed in hospitals that were undergoing extensive restructuring. Results of structural equation analyses showed that workload was positively related to emotional exhaustion. Emotional exhaustion led to cynicism and somatization, and cynicism was negatively related to nurses’ professional efficacy [8].

Within this population, burnout was usually also associated with a mismatch of expectations of a successful medical career and an uncomfortable reality [9]. Globally, 30–50% of clinicians have burnout symptoms, as do about 40% of physicians in primary care positions and more than 50% of specialists in intensive care units. The official prevalence of the syndrome itself is not entirely clear and exact, but extended working hours have been shown to carry a 3% increased risk, with each additional working hour/week, night work or weekend calls adding 3–9% for each additional night or weekend shift. Time at home spent on extra work brings a 2% increased chance for each additional hour/week, while the gap between work and family increases by 200–250% the chances of developing this syndrome [10]. In addition to age, the prevalence of burnout syndrome is also influenced by gender and marital status, but also the age and number of children in the employee’s family. Dissatisfaction with work, overburdening with a large number of patients and the necessary administration are just some of the many stressors that accelerate the onset of burnout [11,12].

In a changing social and physical environment, the brain and body react physiologically and behaviorally to adapt [13,14]. In the physiological state, the organism functions by accelerating adaptation through allostasis. However, constant adaptation and protection of the organism by establishing balance of the body and psyche can lead to overload, then the mechanisms that protected the organism start to work the opposite way and create pathological changes [15,16]. Stress has its main effect on the hypothalamic–pituitary–adrenal gland system, which leads to disorders of cortisol, epinephrine and noreprinephrine levels (hormones regulated by this coupling) [17,18].

On the other hand, a pandemic is rarely a very harmful situation for a whole society, from the work environment to the economic, or one with global consequences.

The new situation affects all sectors of society, slowing them down, but also completely blocking certain activities [19,20,21,22,23]. Certainly, the health system has been hit the hardest. The sudden increase in the number of patients requiring hospitalization has led to a reorganization of the entire system, from the redistribution and additional employment of medical staff to the rearrangement of less busy wards into space for patients infected with the coronavirus and, above all, the rational, but also creatively deployed use of available resources. In addition, a pandemic is a long but dynamic event that requires almost constant adjustment, development of new strategies and problem solving [24,25,26]. In addition, on a global level, the medical system and scientists have had to work extremely hard to discover the key properties of the coronavirus, the changes it leads to, but also prevention in the form of new vaccines. There were 27,861 articles published and indexed in the PubMed database with the keyword “COVID” by 5 July 2021.

The coronavirus pandemic, which has claimed more than 3 million lives so far, has further affected the mental health of people, especially health workers (HW) [27]. In addition to physical risks, it has caused high levels of psychological stress on HW who live in constant fear of exposure to disease, separated from their families, and faced with social stigma [28]. In order to ensure the smooth functioning of medical systems, it is necessary to ensure the well-being and emotional stability of HW, by taking certain measures for the prevention and treatment of any burnout syndrome.

The main purpose of this research was to examine the frequency of burnout syndrome in different health workers such as physicians, pharmacists and nurses employed in the central territory of the Republic of Serbia during the coronavirus pandemic. In this analysis, we will examine some of the aspects of the COVID-19 pandemic on the health system in the Republic of Serbia.

## 2. Material and Methods

### 2.1. Design of Study

This research was a cross-sectional observational questionnaire study of 128 health workers in the central part of the Republic of Serbia. The study surveyed health workers (physicians, pharmacists and nurses) who worked during peaks of the COVID-19 pandemic in the Republic of Serbia at June and November 2020.

### 2.2. Setting and Participants

Inclusion criteria were voluntary permission from each participant, a history of working in the health services at least six months, written and informed consent, age above 21, and duration of work experience more than two years. Exclusion criteria were filling out questionnaires incompletely, a history of working in the health system for less than six months, presence of malignity and psychiatric disorders in history, huge emotional stress in the past six months and intention to change or active job search. The questionnaire was sent electronically to email addresses of health professionals in the period from 22 June 2020 to 15 December 2020, with a break in the period during September due to the increased number of vacations and the calming of the pandemic. The questionnaire was sent to 170 addresses and was completed by 130, and 2 respondents were excluded due to incomplete questionnaires.

### 2.3. Ethical Concerns

From the authors of the questionnaire, permission was obtained electronically for use in this research related to online questionnaire completion. Respondents completed the questionnaire on a voluntary basis, with informed consent and anonymity. The researchers did not have an insight into the identity of the respondents; it was not possible to see the email address of the respondents via a Google link, which ensured complete anonymity and impartiality in the study.

Along with the link to access the survey, respondents were provided with a brief explanation of the purpose of the survey, as well as information on data confidentiality. Since it was a survey of health care professionals after daily work obligations, not patients, an answer itself was understood as implicit consent to participate in the survey.

### 2.4. Maslach Burnout Inventory–Human Services Survey (MBI–HSS–MP] for Medical Personnel

Maslach Burnout Inventory–Human Services Survey [MBI–HSS) adapted for Medical Personnel is a tool for measuring burnout syndrome, as defined by the WHO in the Eleventh International Classification of Diseases. A license from the Mind Group and authors of this questionnaire was obtained for a period of three years (license number 45369) [29].

The MBI-HSS-MP is validated by the extensive research that has been conducted in the more than 35 years since its initial publication. It consists of 22 questions related to the feelings of professionals and their interaction with patients/recipients of services divided into three domains. Each question has 7 possible answers that are scored according to the Likert scale from 0 to 6, with 0 corresponding to the feeling “never” happening and 6 indicating that it happened “every day”. The MBI-HSS-MP addresses three scales (Table 1): (a) emotional exhaustion (EE) measures feelings of being emotionally overextended and exhausted by one’s work; (b) depersonalization (DP) measures an unfeeling and impersonal response toward recipients of one’s service, care treatment, or instruction; and (c) personal accomplishment (PA) measures feelings of competence and successful achievement in one’s work. Demographic data were collected by five demographic questions (age, gender, years of experience, work unit, education and occupation). Designed for professionals in the human services, the MBI-HSS-MP is appropriate for respondents working in a diverse array of occupations, including nurses, physicians, health aides, social workers, health counselors, therapists, police, correctional officers, clergy and other fields focused on helping people live better lives by offering guidance, preventing harm, and ameliorating physical, emotional or cognitive problems

### 2.5. Statistical Analysis

The study sample was calculated according to the assumption of a margin error of 5% and a confidence interval of 95%. Using the online Raosoft sample size calculator (http://www.raosoft.com/samplesize.html, last time accessed: 25 April 2020.), a sample of 134 respondents was calculated, which was rounded down to 130 participants. The questionnaire was sent to 170 addresses and was completed by 130. Two respondents were excluded due to incomplete questionnaires. For the final analysis we included matched 128 participants.

All data were statistically processed using IMB SPSS 26.0 for MacOS. First, the total frequency of answers for each question was processed individually, taking into account the answers of all 128 health workers who filled out the survey. Then, specific groups of respondents were made in order to make a comparison within the examined population. Subsequently, the division of questions was performed on the basis of domains, and then the answers were scored according to the Likert scale, on the basis of which a final conclusion was made about the prevalence of burnout syndrome within the examined population. The chi-square test or Mann–Whitney U test or Fisher’s exact test was used to obtain the initial results and the results of the divided groups, while the collection of Likert scale points was performed in Microsoft Excel 2016 for MS Windows. The chi-square test or linear regression model was used to analyze the influence of covariates on the presence of burnout syndrome.

## 3. Results

The response rate was high, 98%, and 2 of the 130 participants did not give a response on all items, so we included a total number of 128 participants in the analysis.

Validation data confirmed validity and consistency of the questionnaire within the Serbian study population. All three subscales showed high internal consistency with Cronbach’s α coefficient values of 0.821, 0.811 and 0.871 and test–retest reliability was high (*p* < 0.001). The MBI-HSS-MP with 22 questions is a valid and a reliable instrument to assess the burnout status among health workers in Serbia.

In the Table 2 are presented basic demographic characteristics of health workers. Most participants were women of middle age and sufficient work experience in different departments of the health system (Table 2).

Results from the MBI-HSS-MP are presented in Table 3. Significant differences between groups of health workers were observed in the second domain, depersonalization (*p* = 0.014). In other domains, emotional exhaustion and personal accomplishment, there were not observed significant differences between physicians, pharmacists and nurses (Table 2).

In Table 4 is presented prevalence of burnout among physicians, pharmacists and nurses during the COVID-19 pandemic. We observed a high level of emotional exhaustion and personal accomplishment in all tested groups of health workers and a high level of depersonalization in the group of pharmacists (Table 4).

The linear regression model confirmed significant influence of gender and years of experience on emotional exhaustion, as well as influence of occupation on depersonalization. On the other hand, no covariables influenced feelings of personal accomplishment among health workers during the COVID-19 pandemic in the Republic of Serbia (Table 5).

## 4. Discussion

The main goal of this research was to examine the frequency of burnout syndrome in the population of health workers employed in the territory of the Republic of Serbia during the COVID-19 pandemic. Given the high prevalence of this syndrome among employees whose profession is based on working with people, but also the already evident effects of coronavirus on the mental health of the entire population, it was important to examine the current mental state of health workers to take possible treatment and prevention measures, thus preventing the serious consequences of overwork.

As a metaphor for depleting energy, “burning” refers to extinguishing a fire or extinguishing a candle [30,31]. This means that a fire cannot burn continuously unless there are sufficient resources to constantly replenish it. Over time, employees who experience burnout lose the ability to make intensive contributions and from their own perspective or the perspective of others achieve less performance, while continuing to work brings results that are like smoldering. Thus, people used this metaphor to describe their negative work experience before scientific psychology identified it as a phenomenon worth studying [32,33].

The study of burnout syndrome during the pandemic was chosen as the subject of study both because of the popularity of the topic due to the inclusion of the biggest problem of today, and because of the general importance of human mental health, which is often neglected [34,35]. The target group of this research, made up of different profiles of health workers, represents the most vulnerable population for the development of burnout syndrome during a pandemic. Overtime work due to the lack of human resources in health care institutions, as well as additional responsibility and risk of endangering one’s own health, but also the health of the closest family members, undoubtedly placed them in this category. Since our research included a heterogeneous group of health professionals, characteristic groups were singled out, relying on data from the already mentioned papers on the variation of the prevalence of burnout syndrome depending on the characteristics of the respondents themselves [36].

First of all, it is important to emphasize that the incidence of burnout syndrome has been growing in the past few decades and that health workers were one of the most vulnerable populations even before the catastrophe caused by the coronavirus pandemic [37]. There are also studies that show completely different results. The survey conducted in 2018 on nurse-technicians employed at the level of tertiary health care had as the most significant results a low degree of emotional exhaustion (65.7%) and depersonalization (85.7%) of most respondents, while 42.9% had a high a sense of personal achievement [35,38]. However, most studies highlight the high levels of workload of health workers and the drastically increased rate of burnout syndrome since the beginning of the COVID-19 pandemic, with special emphasis on increasing the incidence of burnout in workers who were in direct contact and provided necessary care to infected patients. A study conducted in Italy during the first peak of the pandemic showed that more than 1 in 3 health workers who worked on the first line of defense against the coronavirus had a high level of emotional exhaustion, more than 1 in 4 a high level of depersonalization, but only 15% reported a low sense of personal achievement [15,39,40].

### 4.1. Emotional Exhaustion

To the majority of questions that characterize the domain of emotional exhaustion, and concerning the feelings of health workers towards their work and work atmosphere, the highest percentage of answers was “several times a month” at an average of 24.5%, followed by “18.2%” at “several times a week“. Significant differences can be observed between the emotional exhaustion of physicians and nurse-technicians in relation to masters of pharmacy and pharmaceutical technicians. A higher percentage of masters of pharmacy and pharmaceutical technicians had a high level of emotional exhaustion of 70.3% compared to the comparison group in which this number was 61.5%. This score may be related to the pandemic itself. The changes in the health system that this catastrophe has led to have changed both the scope of work and the responsibilities of many health workers. Doctors and nurse-technicians employed at the levels of primary health care were withdrawn into the COVID systems, while patients were left with limited opportunities to attend regular examinations, which until then had been routinely performed by their chosen doctors. Employed in the domain of the pharmacy institution, therefore, were the only health care workers who remained always available, so patients sought advice on an even larger scale there.

Great emotional exhaustion can be associated with the appearance of skin lesions, and reduced personal efficiency and possibility of adaptation, as well as less social interest in the environment, but also in the family. It is worrying that such disorders, if not recognized and treated in time, can develop into serious mental and somatic diseases over time, which further draws attention to the fact that such high percentages of high emotional exhaustion of health workers should not be ignored [41].

### 4.2. Depersonalization

When it comes to depersonalization as a domain of burnout syndrome, it should be noted that it represents a segment related to relationships with people, in this case most often with patients. According to medical ethics, it can therefore be said to be the most important part of this research. Considering the percentage answers to questions related to the domain of depersonalization, by far the largest percentage of the surveyed population answered “never”, an average of 46.4%, which was scored with 0 points according to the Likert scale, and ultimately affected the low degree of depersonalization. Such data are encouraging because they lead to the conclusion that most health workers, despite the additional risk and stress caused by the new way of working due to the coronavirus pandemic, treat their colleagues and patients with sufficient empathy.

In our study, the largest number of pharmacists showed a high degree of depersonalization. Similar to the results obtained in our study, another study conducted in late 2020 indicated that 25% of pharmacists said that their ability to connect with colleagues and patients decreased during the COVID-19 pandemic, which means that during this period there was a significant increase in depersonalization and that most of these respondents had a high score within this domain [42]. Pharmacists and pharmaceutical industry workers during the COVID-19 pandemic increased their work and sales by about 13% during 2020 in comparison with 2019. The fear-enhanced pandemic has led to a significant increase in demand for drugs and preparations and more visits of patients to pharmacists. In the Republic of Serbia, most supplements and over-the-counter preparations are available on demand without a prescription from a physician, so in case of an increased number of infected patients, first aid was requested at the pharmacy and from pharmacists [43].

### 4.3. Personal Accomplishment

According to the analysis of the total percentages of answers to each question, it is interesting to point out that the question number 19, which reads “I have achieved significant results in this work” has very different answers unlike other questions and the overall score of this domain. The most common answer to question 19 was “several times a month”, while for others the most common answer was “daily” with an average of 37%. The range of the total score of this domain was from 12 to 42; 12 out of 128 respondents (9.4%) had a low level of feelings of personal achievement, 27.3% had a moderate level and 63.3% had a high level. In relation to the general score of burnout syndrome, these data contribute to the reduction of the degree of combustion.

Specifically, the high score for emotional exhaustion and depersonalization is directly proportional to the degree of burnout, while the score of the domain of feelings of personal achievement is inversely proportional; i.e., the higher the feeling of personal achievement, the lower the level of burnout. Consequently, the overall score of a high burnout rate of all subjects was slightly less than 30%, a moderate burnout rate was present in 28%, while a low burnout rate applied to 42% of the population covered by this study.

Based on these results, it can be concluded that the incidence of burnout syndrome calculated on the population included in our study of health workers employed in the Republic of Serbia during the coronavirus pandemic is 58%, taking into account the high and moderate burnout rates. It is difficult to have an exact estimation of the incidence of burnout among physicians, pharmacists and nurses. A recent systematic review including 182 studies published between 1991 and 2018 and involving 109,628 individuals in 45 countries observed a substantial variability in prevalence estimates of burnout among physicians, ranging from 0% to 80.5%. This appeared to be related to important differences in definitions of the syndrome and of the assessment methods applied. Approximately one in three physicians is experiencing burnout at any given time in general [43].

Pharmacists were definitely the most burdened during the COVID-19 pandemic in our country. Some of the reasons are the following: pharmaceutical workers are defined as the first line for calls to health workers, thanks to their availability [44,45,46,47]. They can be described as health care workers at the primary health care level. They work as intermediaries between doctors and patients, and often they themselves participate in giving advice for the treatment of a certain condition without prior appointment. However, the potential of this position, as the first and most frequent point of contact between patients and health care professionals, is relatively underutilized. Despite attempts to strengthen the profession through a vision of even greater commitment to the patient, today the perception of the pharmaceutical profession is increasingly threatened. The reason for that is the growing presence of certain medicines in supermarkets, as well as the development of information technology, which has allowed the online purchase of even medicines and medical devices and supplements. This way of buying pharmaceutical products reduces the authority of pharmacists and pharmacist-patient communication, which can lead to unnecessary complications due to improper use of drugs and supplements [48,49,50].

### 4.4. Study Limitations

One study limitation is related to design of the study, which was cross-sectional, but bearing in mind that a pandemic is a sudden occurrence that lasts for some time, this is reasonable. Another limitation could be a conflict arising from cultural bias and other personal issues which were not evaluated. Further studies must conduct the same research but at multinational and multi-state levels, to evaluate the real impact of the COVID-19 pandemic on health system and health workers.

## 5. Conclusions

According to the results obtained by analyzing the collected data and comparing it with information from the relevant literature, we can conclude that the most health workers have a high degree of emotional exhaustion, but also a low degree of depersonalization and a high degree of sense of personal achievement. Nurses and physicians had similar answers on the pandemic during their work, but pharmacists had different responses. In general, the COVID-19 pandemic seemed emotionally draining, but it encouraged health workers to have a sense of personal achievement due to work and commitment.

## Figures and Tables

**Table 1:** Domains of the MBI-HSS-MP: emotional exhaustion (EE), depersonalization (DP) and personal accomplishment (PA).

Domains	Number of Questions	Ordinal Numbers of Questions
EE	9	1, 2, 3, 6, 8, 13, 14, 16, 20
DP	5	5, 10, 11, 15, 22
PA	8	4, 7, 9, 12, 17, 18, 19, 21

**Table 2:** Basic demographic characteristics of study population.

Total Number of Participants n = 128	Mean	Standard Deviations
Age (years)	38.95	10.634
Years of experience (years)	12.63	10.820
Gender	Female 94 (74.0%)	Male 33 (26.0%)
Work Department		
Emergency department	22 (17.18%)	
General Medicine	39 (30.46%)	
Pharmacies	40 (31.25%)	
Laboratories	22 (17.18%)	
Dentistry	5 (3.93%)	
Occupation		
Physicians	61 (47.65%)	
Nurses	27 (21.09%)	
Pharmacists	40 (31.26%)	
Education		
High education	69 (53.90%)	
Middle education	59 (46.1%)	

**Table 3:** Results from the Maslach Burnout index in the groups of physicians, pharmacists and nurses depending on the domain.

Measure in Domain	Physicians (*n* = 61)	Pharmacists (*n* = 40)	Nurses (*n* = 27)	
	Mean ± SD	Mean ± SD	Mean ± SD	*p* Value
Emotional Exhaustion (EE)	28.21 ± 8.51	31.32 ± 8.57	30.24 ± 8.85	0.933
Depersonalization (DP)	6.89 ± 5.67	10.26 ± 5.19	6.85 ± 4.611	0.014 *
Personal accomplishment (PA)	28.67 ± 6.65	28.79 ± 5.76	28.82 ± 5.69	0.992

Abbreviations: The base date of the “last 2 weeks” is the day when the participants answered the survey. The survey period was from 1–15 December 2020. *p* value was calculated with Mann–Whitney U test. Statistical value less than 0.05 is marked as asterisk (*).

**Table 4:** Prevalence of burnout in physicians, pharmacists and nurses during the COVID-19 pandemic in domains of the MBI-HSS-MP: emotional exhaustion (EE), depersonalization (DP) and personal accomplishment (PA).

	Physicians	Pharmacists	Nurses
EE > 27	61/61 (100%)	40/40 (100%)	27/27 (100%)
DP > 10	2/61 (3.3%)	39/40 (97.5%)	1/27 (3.7%)
PA > 27	61/61 (100%)	40/40 (100%)	27/27 (100%)

Abbreviations: The base date of the “last 2 weeks” is the day when the participants answered the survey. The survey period was from 1–15 December 2020. *p* value was calculated with the Mann–Whitney U test or Fisher’s exact test.

**Table 5:** Prevalence of burnout depending on variables (age, gender, years of experience and occupation). The linear regression model was used to calculate the influence of the continual variables and the chi-square test in case of categorical variables.

Variables	Emotional Exhaustion (EE)				
	Unstandardized B	Coefficients Standardized Error	Standardized Coefficients Beta	t	*p*
Age	0.297	0.183	0.365	1.622	0.107
Gender	−4.479	1.762	−0.227	−2.542	0.012 *
Years of experience	−0.331	0.180	−0.414	−1.845	0.048 *
Occupation	0.722	1.047	0.061	0.689	0.492
	Depersonalization (DP)				
Age	−0.100	0.112	−0.204	−0.892	0.374
Gender	−1.331	1.080	−0.112	−1.232	0.220
Years of experience	0.035	0.110	0.072	0.315	0.753
Occupation	0.249	0.642	0.035	0.387	0.009 *
	Personal Accomplishment (PA)				
Age	0.044	0.135	0.076	0.326	0.745
Gender	1.037	1.2393	0.075	0.802	0.424
Years of experience	−0.023	0.132	−0.042	−0.178	0.859
Occupation	−0.062	0.768	−0.007	−0.081	0.936

Statistical value less than 0.05 is marked as asterisk (*).

## Data Availability

The data presented in this study are available on request from the corresponding author.
